# A Biomimetic Cement-Based Solid-State Electrolyte with Both High Strength and Ionic Conductivity for Self-Energy-Storage Buildings

**DOI:** 10.34133/research.0379

**Published:** 2024-05-22

**Authors:** Wei Lin, Jiarui Xing, Yang Zhou, Long Pan, Li Yang, Yuan Zhang, Xiong Xiong Liu, Chenchen Xiong, Weihuan Li, ZhengMing Sun

**Affiliations:** ^1^ Jiangsu Key Laboratory of Construction Materials, School of Materials Science and Engineering, Southeast University, Nanjing 211189, China.; ^2^ Key Laboratory of Advanced Metallic Materials of Jiangsu Province, School of Materials, Science and Engineering, Southeast University, Nanjing 211189, China.

## Abstract

Cement-based materials are the foundation of modern buildings but suffer from intensive energy consumption. Utilizing cement-based materials for efficient energy storage is one of the most promising strategies for realizing zero-energy buildings. However, cement-based materials encounter challenges in achieving excellent electrochemical performance without compromising mechanical properties. Here, we introduce a biomimetic cement-based solid-state electrolyte (labeled as *l*-CPSSE) with artificially organized layered microstructures by proposing an in situ ice-templating strategy upon the cement hydration, in which the layered micropores are further filled with fast-ion-conducting hydrogels and serve as ion diffusion highways. With these merits, the obtained *l*-CPSSE not only presents marked specific bending and compressive strength (2.2 and 1.2 times that of traditional cement, respectively) but also exhibits excellent ionic conductivity (27.8 mS·cm^−1^), overwhelming most previously reported cement-based and hydrogel-based electrolytes. As a proof-of-concept demonstration, we assemble the *l*-CPSSE electrolytes with cement-based electrodes to achieve all-cement-based solid-state energy storage devices, delivering an outstanding full-cell specific capacity of 72.2 mF·cm^−2^. More importantly, a 5 × 5 cm^2^ sized building model is successfully fabricated and operated by connecting 4 *l*-CPSSE-based full cells in series, showcasing its great potential in self-energy-storage buildings. This work provides a general methodology for preparing revolutionary cement-based electrolytes and may pave the way for achieving zero-carbon buildings.

## Introduction

Buildings come into being along with the human civilization, providing necessary places for human activities, but also consuming large amount of energy. Nowadays, building-related energy consumption is accounting for up to 40% of global energy consumption, which include stages of raw materials production, construction, and daily operation [1–3]. However, building structures possess external walls and surfaces of tremendous volume, which are directly exposed to natural energy sources, such as wind and solar power [4–6]. Since intermittent power generation from those clean energy (i.e., photovoltaic equipment) is difficult to be directly utilized for daily human indoor activities, it is of great significance to develop the self-energy-storage function of buildings [7,8]. Furthermore, by means of the distribution of smart grids and the conversion of secondary energy, massive exposed building walls for energy storage will provide more stable power system and greatly improve energy efficiency, which even achieve zero-energy building and alleviate the energy crisis [9–13].

Cement-based materials are the main component of exposed building surfaces. However, cements encounter challenges in the development of building energy storage, due to the difficulty in simultaneously exhibiting high electrochemical and mechanical properties. Up to now, researchers have explored the possibilities of employing cement-based materials as electrolytes for energy storage devices. In order to markedly improve the ionic conductivity of cement electrolytes, the prevalent strategy involves the incorporation of cations during the initial material mixing phase, typically by adding substances such as potassium hydroxide (KOH) and sodium sulfate (Na_2_SO_4_). Nonetheless, this method bears pronounced drawbacks: it adversely affects the pore structure of cement, exacerbates drying shrinkage, and ultimately results in a decrease in strength of more than 50% [14]. It is senseless to sacrifice fundamental load-bearing capabilities by excessively enhancing ionic conductivity at the expense of mechanical performance. Researchers have also attempted to compensate for some of the strength loss by incorporating polymers. Polyacrylamide and polyacrylic acid have been demonstrated to possess good compatibility with cement [15,16] and hold the capability to enhance the strength and durability of cement [17]. Unfortunately, the addition of these polymers alongside cations to cement fail to effectively improve both strength and ionic conductivity. A pressing conundrum confronts cement electrolytes, where the pursuit of enhanced ionic conductivity invariably takes a toll on the mechanical performance losses [18–20]. Recently, Chanut et al. [21] developed a carbon-incorporated cement electrodes, but such cements still showcased poor mechanical strength. Therefore, there is a growing scientific and technological demand for more electrochemically active cements without compromising the mechanical properties.

Inspired from nature, organized layered composite materials featuring alternating soft and hard phases, such as the spine of sea urchins [22] and the spicules in sponges [23], have been demonstrated to simultaneously enhance toughness and strength, which was previously considered contradictory. This strategy has been introduced into the realm of cement materials, where the structure of natural wood is employed to transform traditional disordered cement structures into ordered layered porous architectures using an ice-templating method [24]. The cement skeleton retains stiffness, while hydrogels as soft phases are filled into the unidirectional pores between neighboring cement lamellae, providing flexibility to deflect cracks and dissipate energy [25]. Consequently, it achieves a simultaneous improvement in both strength and toughness [24,26–29]. Furthermore, this microstructure is expected to facilitate unimpeded ion transport by the interlayer route, thereby remarkably enhancing the ionic conductivity without compromising the mechanical properties. As for the filling hydrogel electrolytes, the polyvinyl alcohol (PVA)-KOH hydrogel is recognized as a prevalent material. It has high ionic conductivity, superior water retention prowess, and cycling stability, can be a viable filling candidate [30–32]. PVA has also shown commendable interface compatibility with cement [33,34], effectively filling voids and mending fissures. Additionally, compared to other commonly used electrolyte materials such as sulfuric acid and sodium sulfate [35] which may cause corrosion to the cement, KOH brings a stronger alkaline environment and is conducive to the hydration reaction and strength development.

In this work, we propose a layered cement-PVA hydrogel solid-state electrolyte (*l*-CPSSE) for self-energy-storage buildings. The *l*-CPSSE employs a cement matrix to serve as the structural bedrock for the electrolyte, thus supplying the requisite mechanical strength and load-bearing capacity, in which the layered micropores are further filled with fast-ion-conducting hydrogels and serve as ion diffusion highways. Simultaneously, the incorporation of PVA-KOH hydrogel not only enhances the electrochemical performance of cement but also facilitates cement hydration. The *l*-CPSSE boasts an exemplary compressive strength of 26.5 MPa and ionic conductivity of 27.8 mS·cm^−1^, overwhelming most previously reported cement-based and hydrogel-based electrolytes. As a proof-of-concept demonstration, we assemble the all-cement-based solid-state energy storage devices, delivering an outstanding full-cell specific capacity of 72.2 mF·cm^−2^. Notably, a 5 × 5-cm^2^ sized building envelop model is successfully fabricated and operated by connecting 4 *l*-CPSSE-based full cells in series, further demonstrating the feasibility of cement-based supercapacitors in self-energy-storage buildings.

## Results and Discussion

### Fabrication of a cement-hydrogel electrolyte

**Fig. 1.**
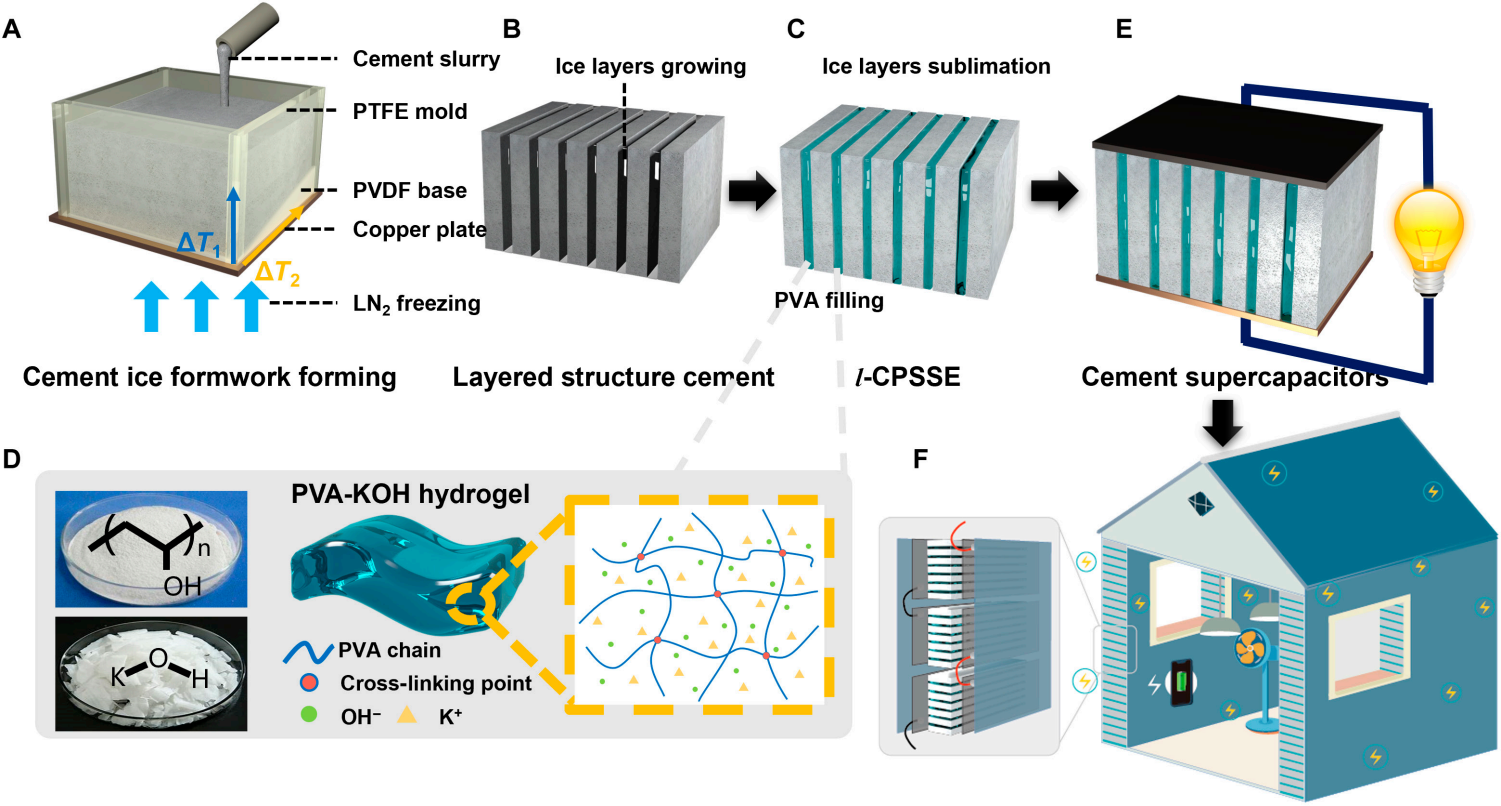
Schematic illustrations of the fabrication process of the layered cement-PVA solid-state electrolyte. (A) The placement of cement slurry in the mold with a bidirectional temperature gradient. (B) The oriented growth of ice sheets and formation of cement lamellae. (C) The filling of PVA hydrogel between the cement lamellae after the ice sublimates. (D) Preparation and structure of PVA-KOH hydrogels. (E) Supercapacitors assembled based on *l*-CPSSE with electrodes. (F) Practical application illustrations of energy storage in building facilities.

As shown in Fig. 1, the fabrication of *l*-CPSSE is conducted in the following 3 steps: Firstly, the cement matrix is prepared by thoroughly mixing cement with water to form a uniform slurry. This slurry is then transferred into a polytetrafluoroethylene mold (Fig. 1A). In order to create a cement structure with oriented pore distribution, the bottom of the mold is brought into contact with liquid nitrogen, leveraging the excellent thermal conductivity of the copper plate within the mold. This contact generates a vertical temperature gradient (Δ*T*_1_) across the mold. An internal polyvinylidene fluoride wedge-shaped insert, having lower thermal conductivity, functions as a barrier between the cement slurry and the copper plate. The difference in thickness of this material generates a horizontal temperature gradient (Δ*T*_2_). Influenced by these vertically bidirectional temperature gradients, the water in the slurry crystallizes into ice layers along the inclined surface, directing the cement particles to positions between these ice layers (Fig. 1B). During the process of sublimation and melting of the ice layers, the cement undergoes gradual in situ hydration, reinforcing the formed layered structure.

The second stage involves the formulation of the hydrogel precursor solution (Fig. 1C and D). PVA and potassium hydroxide (KOH) are dissolved individually in deionized water and then mixed vigorously to obtain the precursor solution of PVA-KOH hydrogel. The cement matrix is submerged in this precursor solution using negative pressure immersion, a simple and effective composite method. By employing the driving force of vacuum negative pressure, the solution permeates the interstitial spaces between the cement layers. Subsequently, freeze–thaw cycles catalyze the transformation of this precursor solution into a hydrogel. *L*-CPSSE is obtained after removing the external excess attached hydrogel.

*L*-CPSSE features a special structure with alternating layers of cement and hydrogel. The cement matrix not only provides strength and mechanical stability but also serves as a carrier for the hydrogel. Simultaneously, the interconnected hydrogel layers facilitate ion transportation, markedly improving the efficiency of ion transfer. When electrode materials are integrated with *l*-CPSSE, a cement-based supercapacitor suitable for energy storage in construction applications is fabricated (Fig. 1E and F). Cement supercapacitors can serve as walls of buildings to store electrical energy for use inside the building. In terms of application security, cement-based supercapacitors should be encapsulated by waterproof coatings and rolls for building exterior and interior walls to prevent ions leakage. The energy storage of building structures can take maximum advantage of cement-based materials in the volume, substantially alleviating the power system load during peak electricity consumption periods. This groundbreaking methodology signals a promising avenue for energy conservation and emission reduction in the construction industry.

### Physicochemical characterizations of the *l*-CPSSE

**Fig. 2.**
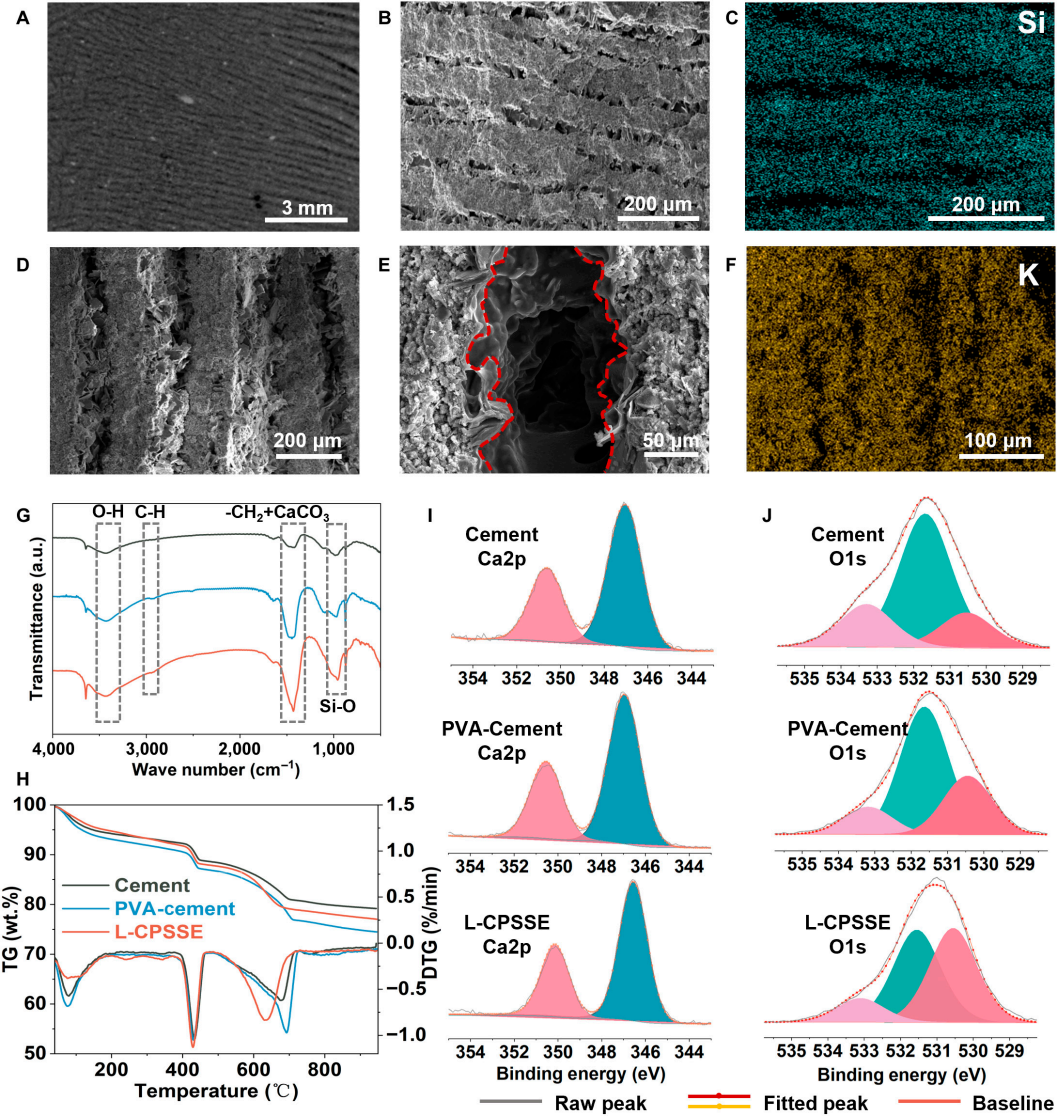
Microstructure and chemical characterization of *l*-CPSSE. (A) X-CT image of the layered cement paste. (B) SEM image of the layered cement paste. (C) EDS mapping pattern of Si in the layered cement paste. (D) SEM image of the *l*-CPSSE. (E) SEM image of the PVA hydrogels connected between cement sheets. (F) EDS mapping pattern of K in the *l*-CPSSE. (G) FTIR patterns. (H) TG and DTG curves. (I and J) Deconvoluted XPS spectra of Ca and O elements of the cement, PVA-cement without KOH and *l*-CPSSE.

As shown in Fig. 2, the structural morphology of the material is characterized using computerized tomography (X-CT), scanning electron microscopy (SEM), and energy-dispersive x-ray spectroscopy (EDS) mapping. The morphological insights garnered from X-CT and SEM imaging reveal that cement presents a well-layered architecture with excellent directional alignment observed consistently across multiple scales during evaluation (Fig. 2A and B). The Si mapping spectroscopy reveals a distinctive layer-by-layer distribution of Si elements, attributed to the enrichment of Si elements in the cement layers during the early stages of hydration (Fig. 2C). This results in a clear distinction between Si-rich layers and interlayer pores within the layered structure cement. There is a substantial accumulation of hexagonal plate-like crystalline calcium hydroxide (Ca(OH)_2_) distributed between the layers, with the introduction of PVA gel (Fig. 2D and E and Fig. S1). The lamellar PVA network bridges the interlayers, adhering to the surfaces of cement particles and hydration products, demonstrating good affinity between PVA and the cement matrix. The K mapping spectroscopy demonstrates a prominent feature of interlayer pore enrichment, along with the presence of potassium elements within the cement layers (Fig. 2F). This can be attributed to the rapid penetration of K^+^ and its adsorption in the capillary pores of the cement layers.

Through Fourier-transform infrared (FTIR) spectroscopy as well as thermogravimetric and x-ray photoelectron spectroscopy (XPS), the influence of PVA hydrogels on cement and the interaction between these 2 materials are characterized. Three sets of samples were tested, including pure cement powder, cement mixed with PVA hydrogel but without KOH, and the *l*-CPSSE composite, which combines cement with PVA-KOH hydrogel. All cement has experienced 5 d of hydration. In the FTIR results (Fig. 2G), the cement electrolytes have a broad absorption band at 3,450 cm^−1^, which is the stretching vibration peak of O–H groups both on the PVA gel and hydrated Ca(OH)_2_. At around 1,400 to 1,500 cm^−1^, the sharper peaks of –CH_2_ stretching vibration from PVA and CaCO_3_ asymmetric stretching from hydration, separately, are evident in the *l*-CPSSE. There is a new peak appeared at 2,930 cm^−1^ in both PVA-cement and *l*-CPSSE samples, which is attributed to the vibration of –CH groups, indicating the successful incorporation of PVA [36]. Furthermore, the content changes of Ca(OH)_2_ and CaCO_3_ can also be exhibited in thermogravimetric results (Fig. 2H), which increase by 6% and 14%, respectively [37]. An enhancement of peak intensity can be observed at 965 cm^−1^ in both PVA-cement and *l*-CPSSE samples, Si–O stretching vibrations of the Q^2^, which is attributed to the characteristic peak of cement hydration product C–S–H. These indicate that the addition of PVA-KOH hydrogels promotes cement hydration reactions, leading to the formation of more hydration products, which is also confirmed by SEM imaging.

XPS characterization is performed on the 3 samples to investigate the chemical states and environments of different elements. In the XPS survey spectrum (Fig. S2), 5 peaks are observed near the binding energies of 102.0, 284.8, 292.9, 347.0, and 531.6 eV, which can be attributed to Si2p, C1s, K2p, Ca2p, and O1s, respectively [38]. The appearance of the new peak at K2p also confirms the successful introduction of KOH-PVA hydrogel. The chemical states of Ca2p and O1s are further analyzed by high-resolution XPS spectra. In the high-resolution XPS spectra of Ca2p (Fig. 2I), the peaks of the cement slurry and PVA slurry completely overlap, while the addition of KOH to PVA results in a decrease in the binding energy of Ca, with a shift of 0.5 eV, indicating a more negative chemical environment for Ca. The peak fitting is performed in the high-resolution XPS spectra of O1s (Fig. 2J), and 3 peaks are identified at binding energies of approximately 530.5, 531.6, and 533.2 eV, and are attributed to silicon hydroxyl, bridging oxygen, and nonbridging oxygen, respectively. Comparing the peak intensities, from cement to PVA-cement and then to *l*-CPSSE, the peak corresponding to nonbridging oxygen gradually increases. PVA hydrogel increases the proportion of nonbridging oxygen, and the addition of KOH further promotes the transformation of bridging oxygen into nonbridging oxygen. PVA and KOH both contribute to the dissociation of Si–O–Si bonds, resulting in shorter silicon chains in the main structure. During this process, due to the presence of lone pairs of electrons and fewer bonding electrons, oxygen atoms become more electronegative, causing a shift in the Ca peak and leading to stronger bonding interactions between Ca and O. Consequently, the connection between the PVA hydrogel and cement layers is enhanced.

### Properties of the *l*-CPSSE

**Fig. 3.**
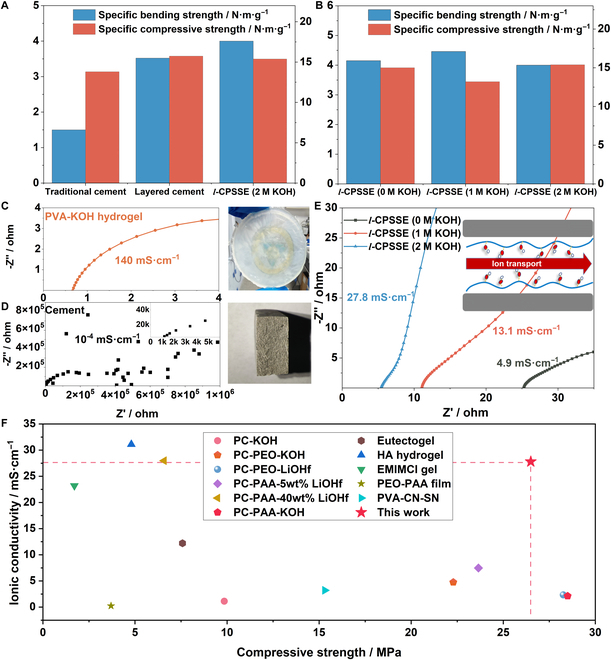
Mechanical properties and ionic conductivity of *l*-CPSSE. (A) Specific bending strength and specific compressive strength of cement prepared by traditional casting, layered cement prepared by ice-template casting, and *l*-CPSSE. (B) Specific bending strength and specific compressive strength of *l*-CPSSE with different KOH concentrations. (C) EIS curve of PVA-KOH hydrogel. (D) EIS curve of cement. (E) EIS curves of *l*-CPSSE with different KOH concentrations. (F) Comparison of strength and ionic conductivity of *l*-CPSSE with other reported SSE.

The mechanical and electrochemical attributes of *l*-CPSSE are fundamental for advancing cement-based supercapacitors. The ice-templating process reduces the density of cement, transforming it into a lightweight and high-strength material. The mechanical properties of traditional cement, layered cement, and *l*-CPSSE with different KOH concentrations are shown in Fig. 3A and B. The cement obtained through the ice template method replaces originally disordered pores in the cement with the unidirectional alignment of microstructures [24,29]. Compared with pores of several millimeters, the pores with tens of micrometers are also beneficial for mechanical properties; therefore, cement produced by ice template method possess higher strengths than traditional method at equal densities. The experimental results of 3-point bending tests indicate that the specific bending strength of the layered cement exhibits a 118% increase compared to the traditionally mixed cement (increasing from 1.6 to 3.5 N·m·g^−1^). Following the injection of pure PVA hydrogel, the specific bending strength of cement illustrates a substantial further improvement, reaching 4.2 N·m·g^−1^, by virtue of the good phase compatibility and stable interfacial connection between the 2 phases, which is consistent with the XPS result. The w/c ratio plays a decisive role in determining the mechanical properties of the electrolyte, as exhibited in Fig. S3A and B. Under each w/c ratio, there is a similarly obvious increase of the specific flexural strength for ice-template cement filled with PVA hydrogel.

As mentioned in FTIR and XPS results, the addition of KOH can promote hydration and strengthen the interface, which is beneficial for strength. However, an excessively high pH value can dissolve calcium ions and hold a negative effect on the microstructure. In a result, the electrolyte exhibited similar compressive and flexural strength within the concentration range of 0 to 2 M. Among them, the specific flexural strength has a slight fluctuation of 6% (4.0 to 4.5 N·m·g^−1^), while for the specific compressive strength it is less than 10% (13.2 to 15.4 N·m·g^−1^). Moreover, regarding the substantial decrease in density, both the unfilled layered cement slurry and *l*-CPSSE exhibit higher specific compressive strength compared to traditionally mixed cement.

The ionic conductivity of PVA-KOH hydrogel, cement, and *l*-CPSSE with different KOH concentrations are evaluated through electrochemical impedance spectroscopy (EIS), as shown in Fig. 3C to E. The PVA-KOH hydrogel exhibits an ultrahigh ionic conductivity of 140 mS·cm^−1^ due to its internal abundance of aqueous phase and expansive gel phase. On the contrary, cement without additions possesses a complex pore structure and low free water content, leading to humongous impedance for ion migration and a low conductivity of only 10^−4^ mS·cm^−1^. After being composite with the hydrogel, *l*-CPSSE experiences a substantial increase in ionic conductivity and is notably influenced by the concentration of KOH. The ionic conductivities of *l*-CPSSE without KOH, with 1 M KOH, and with 2 M KOH are 4.9, 13.1, and 26.7 mS·cm^−1^, respectively. This dramatic improvement is due to the formation of a localized network by the hydrogel within the material and the introduction of a large number of freely mobile foreign ions through KOH. Additionally, *l*-CPSSE without KOH also exhibits relatively good conductivity, indicating that the hydrogel can dissolve and adsorb some of the ions from the cement itself, thereby enhancing the ion conduction ability. Due to the negligible impact of KOH concentration on the mechanical performance, but its direct correlation to the ion conductivity, the subsequent device assembly will involve *l*-CPSSE with a KOH concentration of 2 M for subsequent preparation.

In Fig. S3F, a w/c ratio of 0.8 bolsters the ionic conductivity of *l*-CPSSE, attaining 31.7 mS cm^−1^. The increase in the w/c ratio of ice template cement means more formation of ice crystals and in the spacing between cement layers. With the insertion of hydrogels instead of the original pores, there is a higher hydrogel composition, but reducing mechanical strength (Fig. S3D). Conversely, at a w/c ratio of 0.4 (Fig. S3C and E), *l*-CPSSE exhibits high specific bending and compressive strengths. However, a low w/c ratio prompts the constriction of interlayer pores, hindering the dispersion of the hydrogel precursor solution across the cement layers. This scenario reduces the optimal incorporation of the hydrogel, leading to compromised ionic conductivity. To harmonize the mechanical and electrochemical attributes, a w/c ratio of 0.6 emerges as the preferred choice for subsequent supercapacitor assemblies.

As shown in Fig. 3F, as a cement-based solid-state electrolyte material, *l*-CPSSE exhibits both high mechanical performance and excellent electrochemical characteristics. Within a comparable strength range, the ionic conductivity of *l*-CPSSE surpasses that of most previously reported solid-state electrolyte materials, including aqueous organic electrolytes and cement electrolytes [39–42]. For instance, Zhou et al. [43] designed a layered structure solid-state electrolyte by in situ polymerization of cyanide-based materials combined with PVA. Although their mechanical strength is slightly lower than that of *l*-CPSSE, the ionic conductivity is lower by approximately 2 orders of magnitude. Moreover, *l*-CPSSE even outperforms some quasi-solid-state electrolyte materials. Such materials are typically based on gel matrices [44–47]. Their mechanical performance is comparable to that of *l*-CPSSE, but their conductivity at room temperature is still lower by an order of magnitude. Meanwhile, within a similar range of conductivity, *l*-CPSSE maintains a marked advantage in terms of mechanical performance [48,49]. In comparison to a similar cement-based electrolyte, Zhang et al. [17] prepared PAA-LiOHf-Cement electrolyte, achieving an ionic conductivity of 27.97 mS/cm by doping control, with a compressive strength of less than 7 MPa, which is lower than *l*-CPSSE. It is crucial to emphasize that *l*-CPSSE addresses the challenge of enhancing the comprehensive performance of solid-state electrolytes, offering insights into improving the performance of cement-based energy storage devices and bringing promise for practical applications.

### Electrochemical performance of structural supercapacitors

**Fig. 4.**
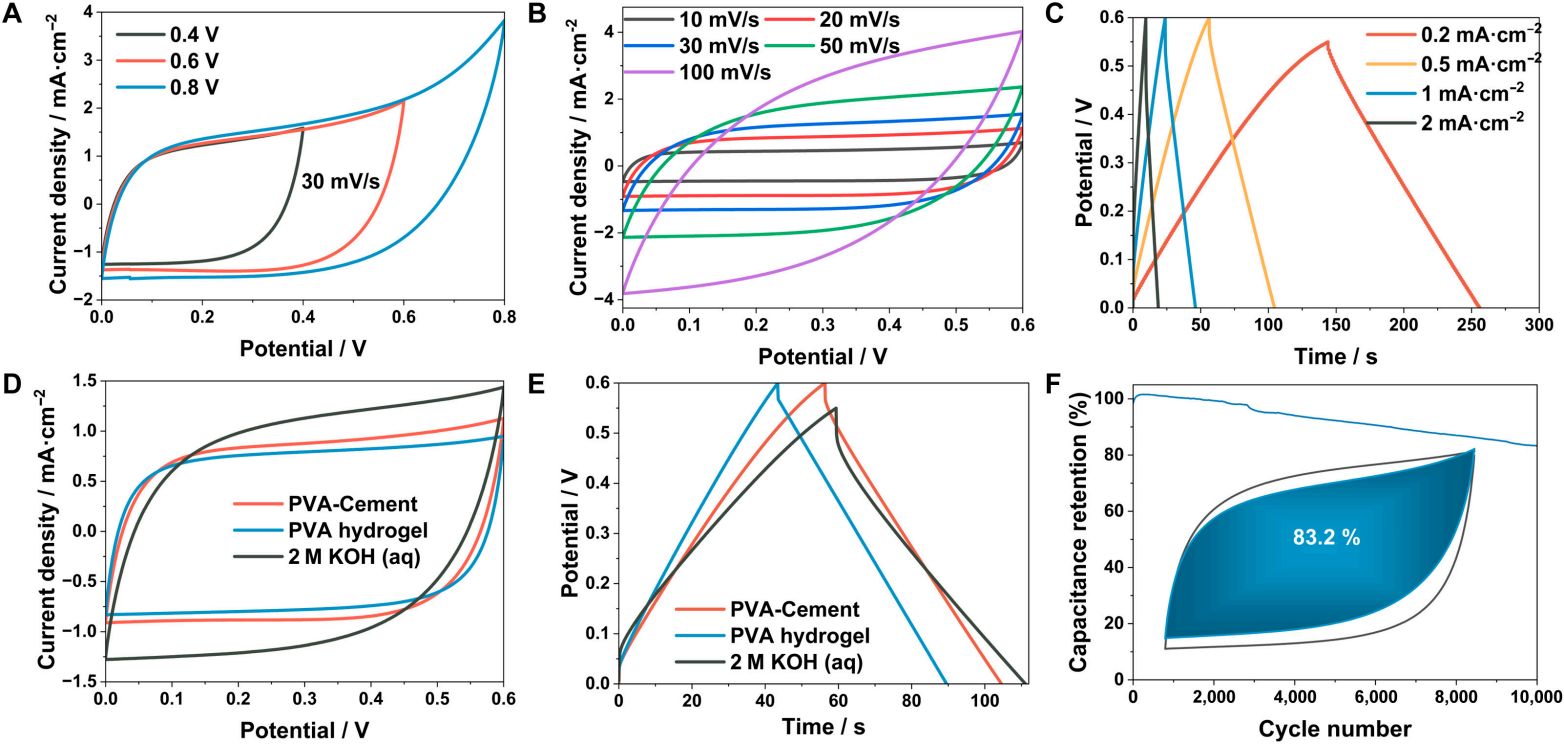
Electrochemical properties of supercapacitors assembled by *l*-CPSSE and Ti_3_C_2_T_x_ electrodes. (A) CV images of cement supercapacitors in different voltage ranges. (B) CV images of cement supercapacitors at different sweep speeds. (C) GCD images of cement supercapacitors at different current densities. (D) CV image comparison of cement supercapacitor, PVA-KOH hydrogel supercapacitor, and 2 M KOH (aq) supercapacitor. (E) GCD image comparison of cement supercapacitor, PVA-KOH hydrogel supercapacitor, and 2 M KOH (aq) supercapacitor. (F) Specific capacity changes of cement supercapacitors in long cycle test.

The practical application of *l*-CPSSE is demonstrated through the electrochemical performance of cement supercapacitors assembled with Ti_3_C_2_T_x_ electrodes. The cyclic voltammetry (CV) curves at different voltage ranges are compared in Fig. 4A, which shows dramatic polarization phenomena at the voltage of 0.8 V. The electrochemical stable voltage window of the device is determined to be 0.6 V, showing good electrochemical stability within the range of 0 to 0.6 V. Subsequently, a comparison of CV at various scan rates ranging from 10 to 100 mV·s^−1^ is conducted (Fig. 4B). The CV results exhibit quasi-rectangular curves within the scan rate range of 10 to 50 mV·s^−1^. As the scanning rate reaches 100 mV·s^−1^, the rectangular CV shape distorts to nearly fusiform. This phenomenon might be attributed to the slower inclusion/ejection and diffusion of counterions compared to the rapid electronic transfer in Ti_3_C_2_T_x_ at high scanning rates [50].

The charge–discharge performance of the device at different current densities is evaluated through galvanostatic charge–discharge (GCD) tests (Fig. 4C). The specific capacitance values at 0.2, 0.5, 1, and 2 mA·cm^−2^ are found to be 40.7, 40.5, 38.3, and 31.7 mF·cm^−2^. The symmetric charge–discharge curves further confirm the typical behavior of the electric double-layer capacitance. As reference electrolyte materials, pure PVA-KOH hydrogel and 2 M KOH solution are respectively assembled with symmetrical Ti_3_C_2_T_x_ electrodes in Fig. 4D and E. In the aCV results at a scan rate of 20 mV·s^−1^, the integrated areas are very close and slightly smaller than the aqueous-based supercapacitor, indicating that both have similar energy storage performance. The results from the GCD test confirm this conclusion. The area-specific capacitance of the cement supercapacitor and the hydrogel supercapacitor at a current density of 0.5 mA·cm^−2^ are 40.5 and 39.2 mF·cm^−2^. The equal energy storage capacity shows that in *l*-CPSSE, the cement matrix not only provides structural support and enhances electrolyte stability but also preserves the inherent capacitance behavior of the hydrogel electrolyte, without causing a substantial decline in its electrochemical performance. Finally, a charge–discharge cycle is performed assembled with the *l*-CPSSE at a current density of 2 mA·cm^−2^, and the cement supercapacitor shows a higher capacitance retention of 83.2% after 10,000 cycles at 2 mA cm^−2^ (Fig. 4F). In comparison to some of the previously reported supercapacitors, the cement supercapacitor exhibits outstanding cycling stability [51,52].

### Demonstrations of self-energy-storage buildings

**Fig. 5.**
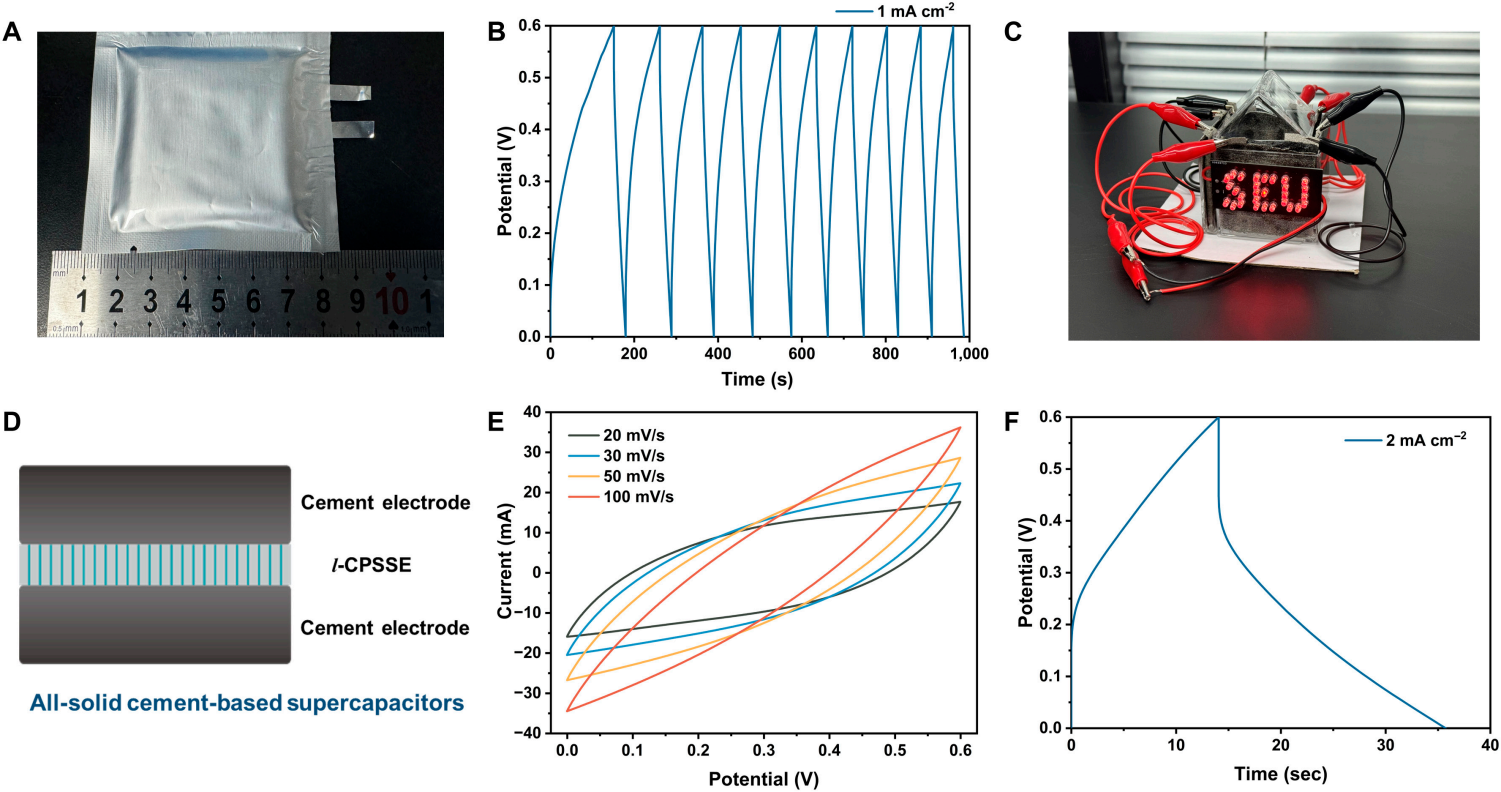
The further demonstration of as-developed cement-based supercapacitors. (A) The photograph of cement supercapacitor with the size of 50 mm × 50 mm encapsulated in the aluminum–plastic film. (B) GCD curve of the pouch supercapacitor at the current density of 1 mA·cm^−2^. (C) The photograph of the light-emitting diode lamp which is powered by a house model (4 cement supercapacitors connected in series). (D) Schematic illustration of the structure of the all-solid cement-based supercapacitors. (E) CV images of all-solid cement-based supercapacitors at different sweep speeds. (F) GCD curve of all-solid cement-based supercapacitors at the current density of 2 mA·cm^−2^.

The practical application of the cement-based electrolyte developed above to self-energy-storage buildings still needs to solve 2 key issues: One is the verification of material size expansion, and the other is the matching of suitable electrode materials. First, through the packaging method of the pouch supercapacitors, the size of *l*-CPSSE has been expanded to 5 cm × 5 cm. As shown in Fig. 5A, the *l*-CPSSE and symmetrical electrodes are encapsulated with aluminum–plastic film. In 10 stable charge–discharge cycles, the cement supercapacitors have an average specific capacity of 38.1 mF·cm^−2^ at a current density of 1 mA·cm^−2^ (Fig. 5B), which is approximately equal to the 38.3 mF·cm^−2^ specific capacity assembled in a button cell casing (Fig. 4C). *L*-CPSSE and symmetrical electrodes mentioned above are assembled as a building wall, and we use 4 walls connected in series to form a self-energy-storage building envelop model, achieving continuous illumination of light-emitting diode lamps (Fig. 5C). Specific steps are as shown in the supplementary movie.

On the other hand, the electrode material that is most suitable for cement-based electrolyte is obviously also cement. Therefore, we prepared cement electrodes containing carbon black, referring to the work of Chanut et al. [21]. It is based on the inherent pore structure of cement-based materials, which adsorbs a large number of potentially connected carbon nanoparticles. An all-cement-based solid-state supercapacitor assembled by *l*-CPSSE is as shown in Fig. 5D. Figure 5E shows the typical CV images of the all-solid cement-based supercapacitors. Within the voltage range of 0 to 0.6V, the symmetrical CV patterns indicate that the device possesses a typical electric double-layer capacitance. Within the scan rate range of 20 to 100 mV·s^−1^, the shape of the curves undergoes no substantial deformation with increasing scan rate. This suggests that the device exhibits low polarization effects in this interval, demonstrating efficient capacitive behavior and excellent charge storage capacity, along with fast charge and discharge kinetics. In the test of GCD (Fig. 5F), all-solid cement-based supercapacitors exhibit a high specific capacitance of 72.2 mF·cm^−2^ at a current density of 2 mA·cm^−2^. The further increase in specific capacitance may be due to better interface compatibility between the electrode and electrolyte, and high adsorption capacity of carbon black.

In order to further increase the capacity, pseudo-capacitors assembled with *l*-CPSSE and Co_3_O_4_ electrodes have also been attempted (Fig. S5), based on differentiated charge storage mechanisms. GCD test results show that the cement supercapacitors assembled by Co_3_O_4_ electrodes respectively represent specific capacities of 187.2 and 157.6 mF·cm^−2^ at current densities of 1 and 2 mA·cm^−2^. Compared to most reported cement-based and hydrogel-based supercapacitors, the capacity of cement supercapacitors developed in this work remarkably increases. The electrolyte materials used in these supercapacitors include PVA-H_3_PO_4_ hydrogel, PVA-H_2_SO_4_ hydrogel, cement, and sulfone-based electrolytes [53–56]. Among them, Yang et al. [57] assembled PVA-H_3_PO_4_ hydrogel with composite electrode materials of carbon nanotubes and polymers to obtain the supercapacitor with excellent mechanical flexibility and tensile properties, but at the current density of 0.1 mA·cm^−2^, the surface capacity is 20 mF·cm^−2^, lower than the cement supercapacitor. Additionally, Zhang et al. [14] assembled a cement-based electrolyte with reduced graphene oxide electrodes to obtain a supercapacitor with a specific capacity of less than 30 mF·cm^−2^ at a current density of 0.1 mA·cm^−2^, which energy storage performance is slightly inferior to the device designed in this work. This observation underscores the favorable electrochemical performance of the cement-based supercapacitors assembled by *l*-CPSSE. Through the expansion of material size, adaptation of cement electrodes, and improvement of device capacity, *l*-CPSSE exhibits the potential for energy storage devices, and we believe that it may pave the way to achieve self-energy-storage buildings.

## Conclusion

In conclusion, this study introduces a cement-based solid-state electrolyte material with an ordered layered structure, as a dramatic approach to the advancement of self-energy-storage buildings. The distinctive architecture of *l*-CPSSE has been validated, showcasing a successful formation of the layered structure of cement and the effective incorporation of PVA hydrogel between these layers. The superior interface compatibility between the hydrogel and the cement matrix is also proved, revealing that the hydrogel plays a pivotal role in catalyzing the cement hydration reaction, which subsequently augments strength development and stability of the composites. Concurrently, the interlayer hydrogels, with their abundant water phase and introduced external ions, remarkably improve the electrochemical performance of the cement-based material. As a result, *l*-CPSSE achieves an impressive 3.4 N·m·g^−1^ of specific bending strength, 16.6 N·m·g^−1^ of specific compressive strength, and a high ion conductivity of 27.8 mS·cm^−1^, outweighing most other solid-state electrolyte materials in consideration of both mechanical and electrochemical properties.

Furthermore, the cement-based supercapacitor, fabricated based on *l*-CPSSE, manifests prodigious energy storage capabilities. Assembled with carbon electrodes, cement electrodes, and Co_3_O_4_ electrodes, the cement supercapacitors obtain specific high areal capacities of 40.7, 72.7, and 187.2 mF·cm^−2^, respectively, and it also exhibits good cycle stability. Notably, the high performance of all-cement-based supercapacitors and successful operation of the building envelop model (connecting 4 *l*-CPSSE-based full cells in series) further demonstrate the application feasibility of *l*-CPSSEs in building energy storage. In the future, under large-scale practice, building cement structures can store renewable energy converted from solar and wind energy, supplying for daily human activities. The unique microstructure of *l*-CPSSE can also be regarded as thermoelectric material which relies on temperature difference to generate heat, unifying ecological power generation and self-energy-storage building. This study proposes a fresh avenue for crafting cement-based energy storage materials, paving the way for innovative strategies aimed at energy conservation and carbon footprint reduction in the construction sector.

## Materials and Methods

### Materials

Ordinary Portland cement (P I 42.5) was offered by United Cement Co. Polydimethylsiloxane was purchased from Dow Corning. PVA (analytical grade, molecular weight 80,000 to 90,000) was purchased from Aladdin reagent. Potassium hydroxide (KOH, 95% analytical purity) was purchased from Macklin.

### Preparation of cement-PVA hydrogel electrolyte

To begin, a cementitious matrix with a layered structure was fabricated using the ice-templating method. Based on gradient water-to-cement ratio of 0.6, ordinary Portland cement was thoroughly mixed with water in a blender, with slow stirring for 1 min followed by fast stirring for 2 min. Subsequently, the cement slurry was poured into custom polytetrafluoroethylene molds. The dimensions of the molds were 50 mm × 50 mm × 50 mm, with a wall thickness of 1cm. An inclined polydimethylsiloxane wedge was placed inside the mold, with an angle of 20 °, connected to a copper plate at the base. Thermal conduction between liquid nitrogen and the molds was facilitated by a copper column with a diameter of 5 cm and a copper plate: the mold was positioned on the copper column, with one end of the column in contact with the copper plate and the other end immersed in liquid nitrogen. After freezing for 1 h in liquid nitrogen, layered cement was initially prepared. Then, the mold was removed and placed in a refrigerator at 5 °C for 1 d to slowly thaw, followed by demolding and subsequent immersion in water for curing.

Regarding the preparation of hydrogel precursor solution, 5 g of PVA was dissolved in 40 g of distilled water with stirring at 98 °C for 1 h. Then, a certain proportion of KOH was dissolved in 5 g of distilled water, which is slowly added to the PVA solution, and the PVA-KOH solution was obtained. The PVA-KOH solution was transferred to a culture dish and immersed in hardened cement blocks. Negative pressure impregnation was used to ensure that the solution penetrated into the interstitial spaces between the cement layers, lasting for 1 h. Then, the layered cement filled with PVA-KOH solution was frozen at −20 °C for 12 h, thawed for 6 h, and repeated 3 times. *L*-CPSSE with the density of 1.8 g cm^−3^ was obtained after removing the external excess attached hydrogel. The *l*-CPSSEs with gradient w/c ratios and different KOH concentrations were prepared by the same method.

### Preparation of delaminated MXene (d-Ti_3_C_2_T_x_) electrode

First, a conventional route was followed to synthesis d-Ti_3_C_2_T_x_. LiF/HCl solution was used to etch Ti_3_AlC_2_ (purified, ≥90 %) to synthesize multilayer Ti_3_C_2_T_x_ (m-Ti_3_C_2_T_x_). Then, delaminated Ti_3_C_2_T_x_ (d-Ti_3_C_2_T_x_, marked as Ti_3_C_2_T_x_) was obtained by ultrasonic treatment of m-Ti_3_C_2_T_x_ under argon atmosphere followed by freeze drying.

Second, to prepare electrode slurry, Ti_3_C_2_T_x_ powder was mixed with Super P (used as conductive agent) and sodium carboxy methyl cellulose (binder) in deionized water to form a homogenous slurry. Then, the slurry was carefully pasted to nickel foams, followed by vacuum-drying under 60 °C for 12 h subsequently.

### Assembly of solid-state supercapacitor

The testing electrodes were prepared by coating Ti_3_C_2_T_x_ onto a foam nickel current collector through solvent evaporation. The mass loading of Ti_3_C_2_T_x_ electrode was 1.6 mg·cm^−2^. The dimension of cement-PVA hydrogel electrolyte and electrodes was approximately 10-mm length × 10-mm width, and the thickness of electrolyte is around 1.5 mm. The solid-state supercapacitor was assembled in a symmetric 2-electrode configuration, sealed within a CR2032 button cell casing. To further demonstrate the feasibility of *l*-CPSSE in building energy storage, we also test the cement-hydrogel electrolyte with the size of 50 mm × 50 mm, encapsulated with aluminum–plastic film. Connecting 4 cement supercapacitors with the size of 50 mm × 50 mm in series, we built a building model, and the detailed procedure was shown in the supplementary movie.

### Microstructure characterizations

The microstructural features of the material were characterized using a field emission scanning electron microscope (SEM 434 FEI 3D) operated at an acceleration voltage of 20 kV. Elemental mapping was performed by scanning the sample surface 16 times using an EDAX elemental analysis system. Before electron imaging, the sample was dried and coated with a thin platinum film using a high-resolution coater. The layered structure and distribution of hydration products in the ice-templated cement were examined using x-ray computed tomography (Zeiss Xradia 510 Versa). The sample was rotated 360 ° about a normal axis relative to the detector, capturing 1,000 to 1,400 2D projection slices. FTIR spectra were obtained in the wave number range of 400 to 4,000 cm^−1^ using a Nicolet iS10 spectrometer. Using a synchronous thermal analyzer Q600 for thermogravimetric analysis (TGA), the test temperature range was from room temperature to 950 °C, and the heating rate was 10 °C/min. The chemical states, chemical environments of each element, and binding energy shifts were characterized using XPS (Thermo SCIENTIFIC ESCALAB 250Xi).

### Mechanical testing

Before testing, the cement-hydrogel composite materials were processed into test specimens with dimensions of 40 mm in length, 15 mm in width, and 8 mm in thickness using a cutting machine and a grinding and polishing machine. ACMT 4503 universal testing machine with a maximum load capacity of 300 kN and a loading rate of 0.5 mm/min was used for 3-point bending tests. The bending load was applied perpendicular to the direction of the bone cement thin slice. For the compressive strength test, a UMT 5105 universal testing machine with a maximum load capacity of 100 kN was employed. The loading rate in the compression test was 100 N/s.

### Electrochemical tests

Electrochemical characterizations, including EIS, CV, and GCD, were conducted using a biological electrochemical workstation. All electrochemical tests were conducted at room temperature. All electrochemical performance value calculations were based off 3-time measurement at least.

When testing ion conductivity, the cement electrolyte was clamped between the steel sheets, and the electrolyte's internal resistance *R*_b_ was calculated from the intercept on the real axis of the EIS curve. Then, the ionic conductivity σ was calculated using Eq. 1.

$$\sigma =\frac{H}{SR_{b}}$$ (1)

Here, *H* and *S* are the thickness and the sectional area of the sample, respectively.

The specific capacitance of the prepared electrode and supercapacitor were calculated based on Eq. 2.

$$C_{F}=\frac{I\times ∆\;t}{A\times ∆\;V}$$ (2)

Here, *I* (mA) is the discharge current, ∆*t* (s) is the discharge time, *A* (cm^2^) is the area of the active material, and ∆*V* (V) is the potential window, respectively.

A 3-electrode system was employed for CV and GCD studies on the synthesized Ti_3_C_2_T_x_ electrode in a 2 M KOH electrolyte. The prepared electrode served as the working electrode, a Hg/HgO electrode acted as the reference electrode, and a platinum sheet electrode was used as the counter electrode.

## Data Availability

The authors declare that the data supporting the findings of this study are available within the paper and its supplementary information files.
