# Supplementary material for: A Biomimetic Cement-Based Solid-State Electrolyte with Both High Strength and Ionic Conductivity for Self-Energy-Storage Buildings
Source: Research (Wash D C). 2024 May 22;7:0379. doi: 10.34133/research.0379 (PMC11109515; doi:10.34133/research.0379)
Supplement: Supplementary 1 — Figs. S1 to S5 Movie S1 [file research.0379.f1.zip › Revised Supplementary file.docx]

**Supporting Information**

**A Biomimetic Cement-Based Solid-State Electrolyte with both High Strength and Ionic Conductivity for Self-Energy-Storage Buildings**

Wei Lin ^a, 1^, Jiarui Xing ^a, 1^, Yang Zhou ^a, *^, Long Pan ^b, *^, Li Yang ^b^, Yuan Zhang ^b^, Xiong Xiong Liu ^b^, Chenchen Xiong ^a^, Weihuan Li ^a^, ZhengMing Sun ^b, *^

^a^ Jiangsu Key Laboratory of Construction Materials, School of Materials Science and Engineering, Southeast University, Nanjing 211189, China

^b^ Key Laboratory of Advanced Metallic Materials of Jiangsu Province, School of Materials, Science and Engineering, Southeast University, Nanjing 211189, China

^*^ Email: [tomaszy@seu.edu.cn](mailto:tomaszy@seu.edu.cn) (Y. Z.); [panlong@seu.edu.cn](mailto:panlong@seu.edu.cn) (L. P.); [zmsun@seu.edu.cn](mailto:zmsun@seu.edu.cn) (Z. S.).

^1^ These two authors contributed equally to this work and should be considered co-first authors.


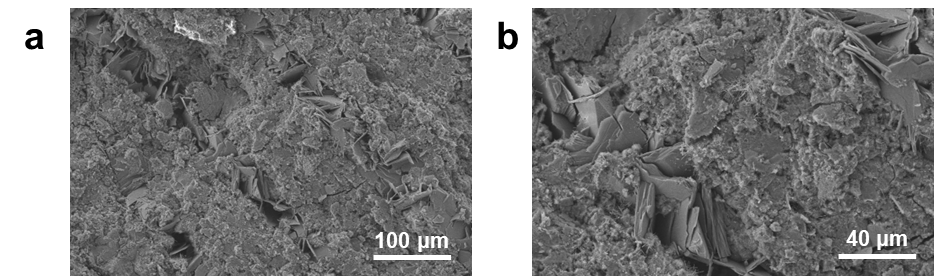


**Fig. S1** SEM images of crystalline calcium hydroxide between the layers. (a) 100 μm; (b) 40 μm.


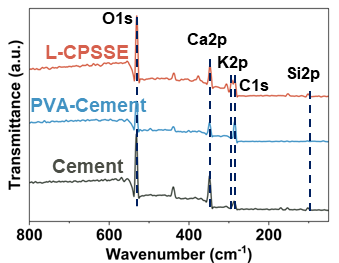


**Fig. S2** Wide region XPS spectra of *l*-CPSSE.


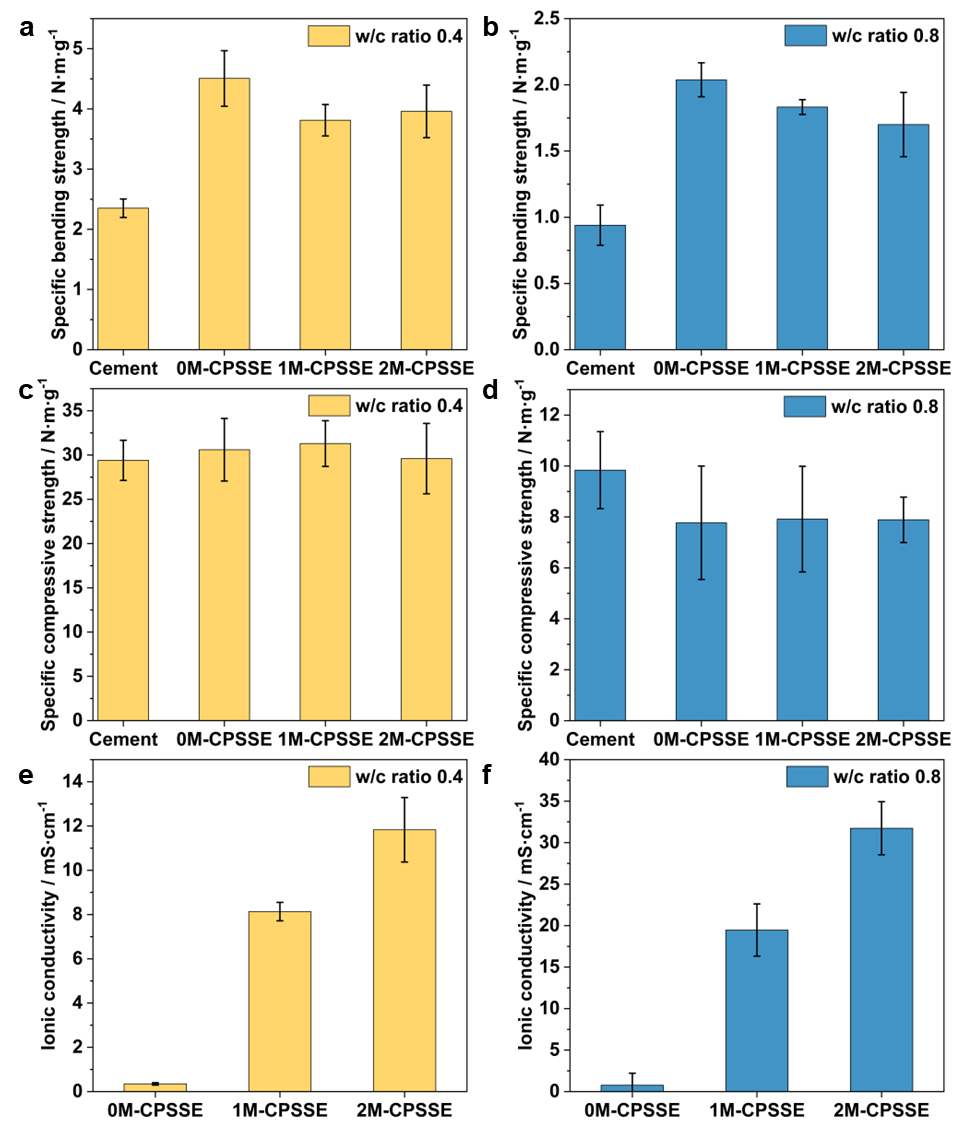


**Fig. S3** Image of property comparisons of traditional cement and *l*-CPSSE with different KOH concentration. (a) Specific bending strength with a w/c ratio of 0.4. (b) Specific bending strength with a w/c ratio of 0.8. (c) Specific compressive strength with a w/c ratio of 0.4. (d) Specific compressive strength with a w/c ratio of 0.8. (e) Ionic conductivity with a w/c ratio of 0.4. (f) Ionic conductivity with a w/c ratio of 0.8.

Fig. S4 shows the electrochemical performance results of the Ti_3_C_2_T_x_ electrodes. The electrode materials are immersed in a 2M KOH solution, and their electrochemical performance is tested using a three-electrode setup. The voltage window for the electrode materials is set at 0.7 V. The specific capacitances are measured at different current densities: 150 F·g^-1^ at 1 A·g^-1^, 142 F·g^-1^ at 2 A·g^-1^, and 114 F·g^-1^ at 5 A·g^-1^.


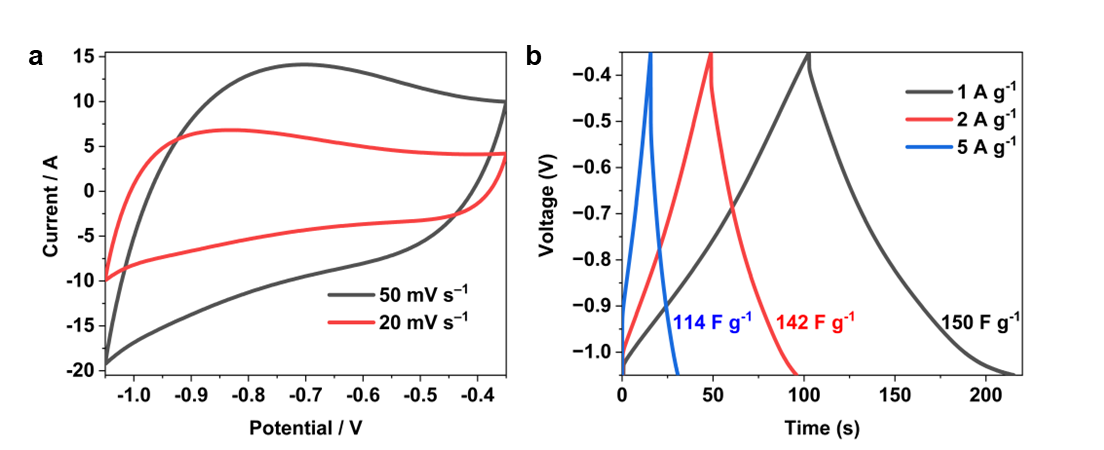


**Fig. S4** Image of test results of Ti_3_C_2_T_x_ electrodes by three-electrode method. (a) CV curve at different sweep speeds. (b) GCD curve and specific capacity at different current densities.

The Co_3_O_4_ electrodes were prepared by a facile hydrothermal synthesis and calcination. First, the Ni foam was ultrasonically cleaned in ethanol and H_2_O for 15 min. Then, 5 mmol of urea nitrate, 2 mmol of ammonium fluoride and 1 mmol of cobalt were dissolved in 10 mL of distilled water. It was transferred into a PTFE-lined stainless steel autoclave. After that, a 2 × 6 cm^2^ piece of cleaned substrate was put into the above solution. After heated at 120 °C for 5 h, it was cooled to ambient temperature. The sample was carefully removed and cleaned, then dried in air at 60 °C for 12 h. Finally, the precursor was calcined at 400 °C for 2 h at a heating rate of 2 °C min in an ambient atmosphere.

The mass loading of Co_3_O_4_ electrode was around 15 mg·cm^-2^. The dimension of cement-PVA hydrogel electrolyte and electrodes was also approximately 10 mm length ×10 mm width. And the thickness of electrolyte is around 1.5 mm. The cement-based solid-state supercapacitors were assembled into asymmetric devices using Co_3_O_4_ electrodes and activated-carbon electrodes, sealed within a CR2032 button cell casing. Electrochemical properties were as shown in Fig. S5.


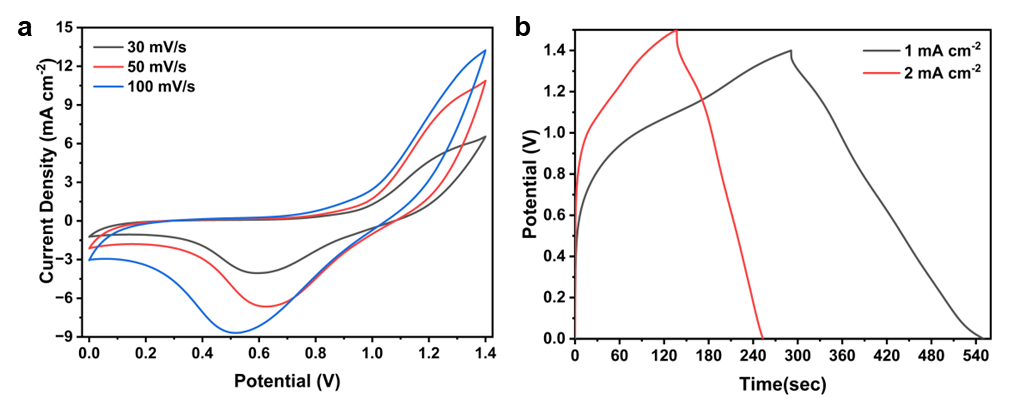


**Fig. S5** Electrochemical properties of cement supercapacitors assembled by Co_3_O_4_ electrodes. (a) CV images of cement supercapacitors at different sweep speeds. (b) GCD images of cement supercapacitors at different current densities.
