# Supplementary figures and images for: A Biomimetic Cement-Based Solid-State Electrolyte with Both High Strength and Ionic Conductivity for Self-Energy-Storage Buildings
Source: Research (Wash D C). 2024 May 22;7:0379. doi: 10.34133/research.0379 (PMC11109515; doi:10.34133/research.0379)

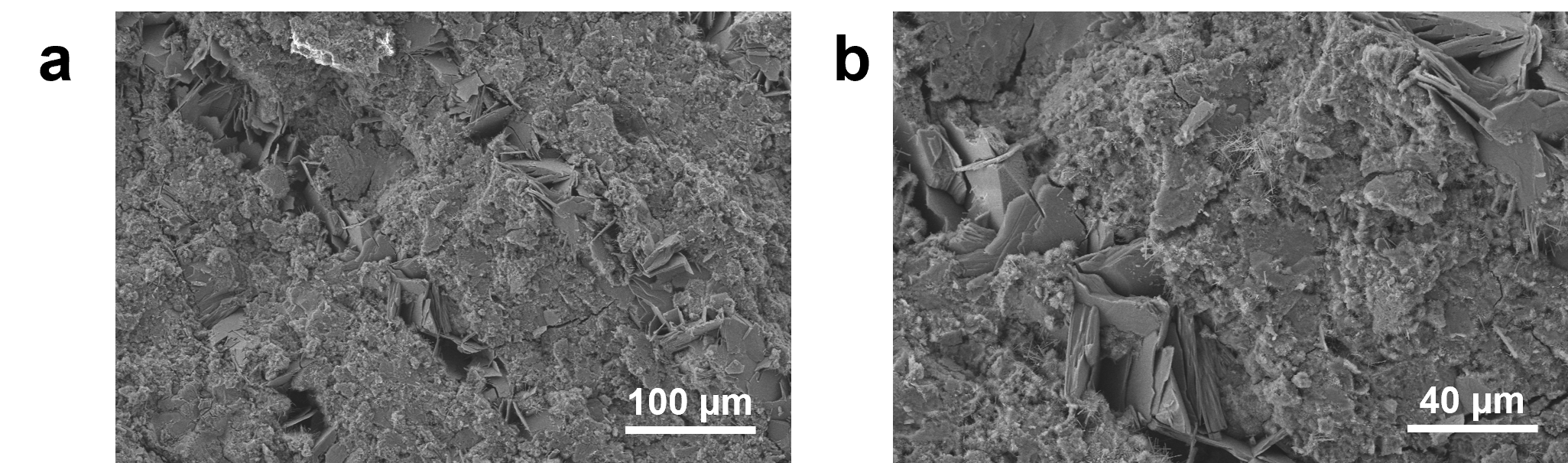

Supplement: Supplementary 1 — Figs. S1 to S5 Movie S1 [file research.0379.f1.zip › Fig. S1.tif]

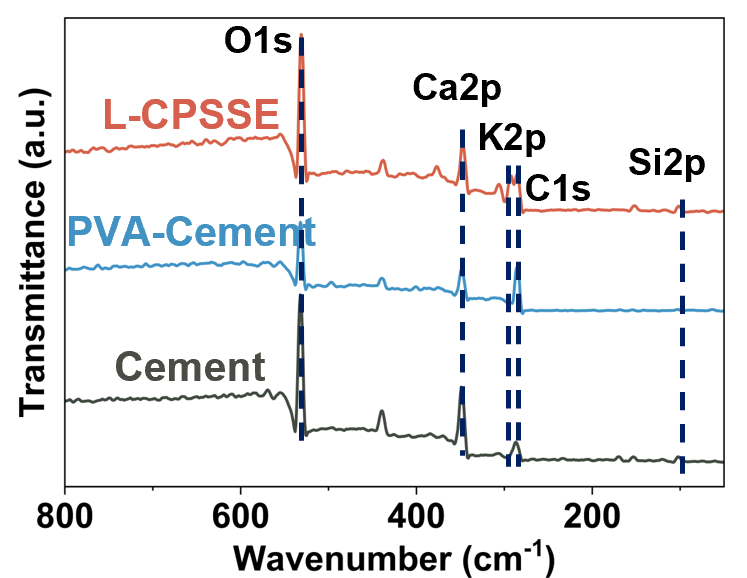

Supplement: Supplementary 1 — Figs. S1 to S5 Movie S1 [file research.0379.f1.zip › Fig. S2.tif]

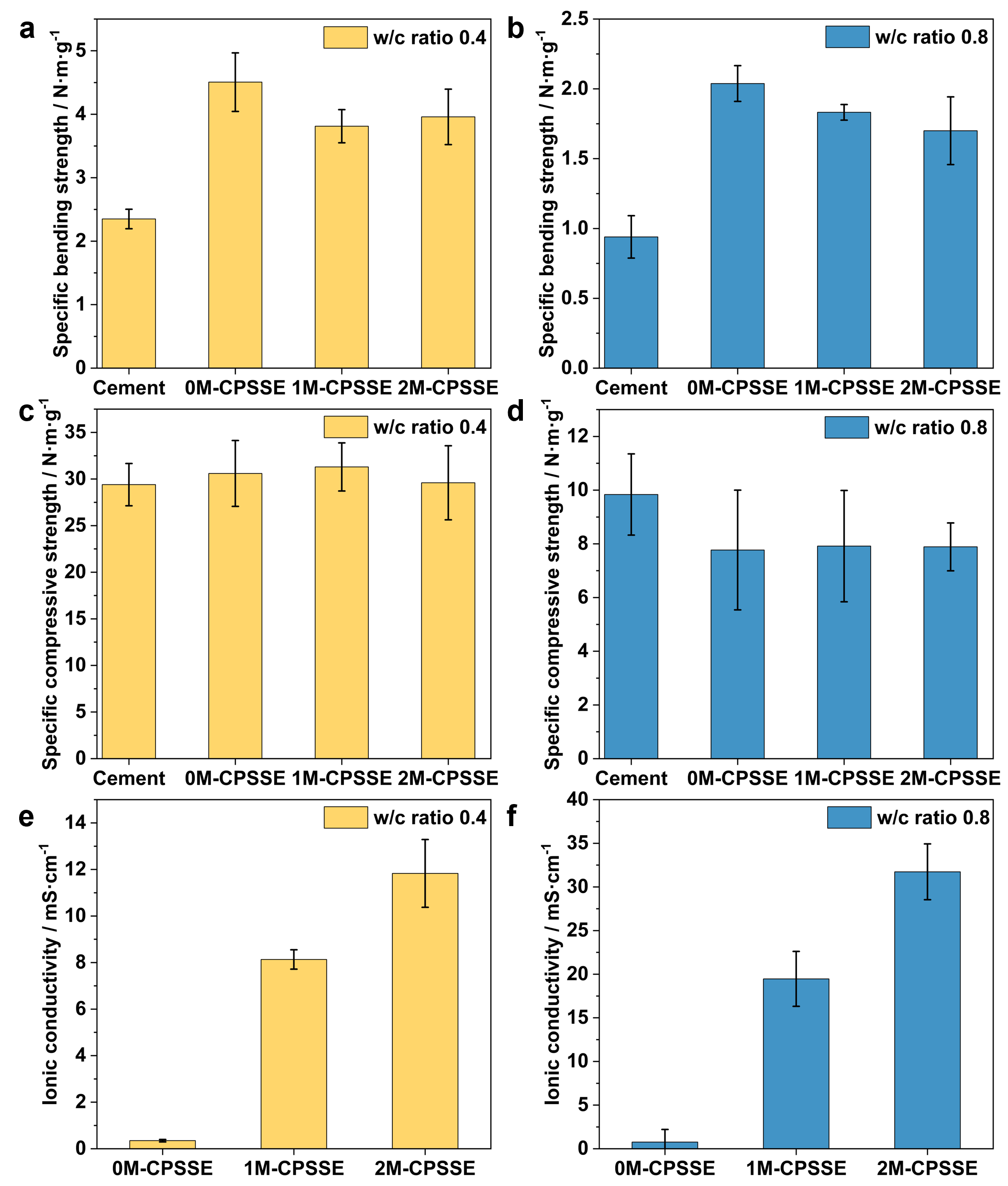

Supplement: Supplementary 1 — Figs. S1 to S5 Movie S1 [file research.0379.f1.zip › Fig. S3.tif]

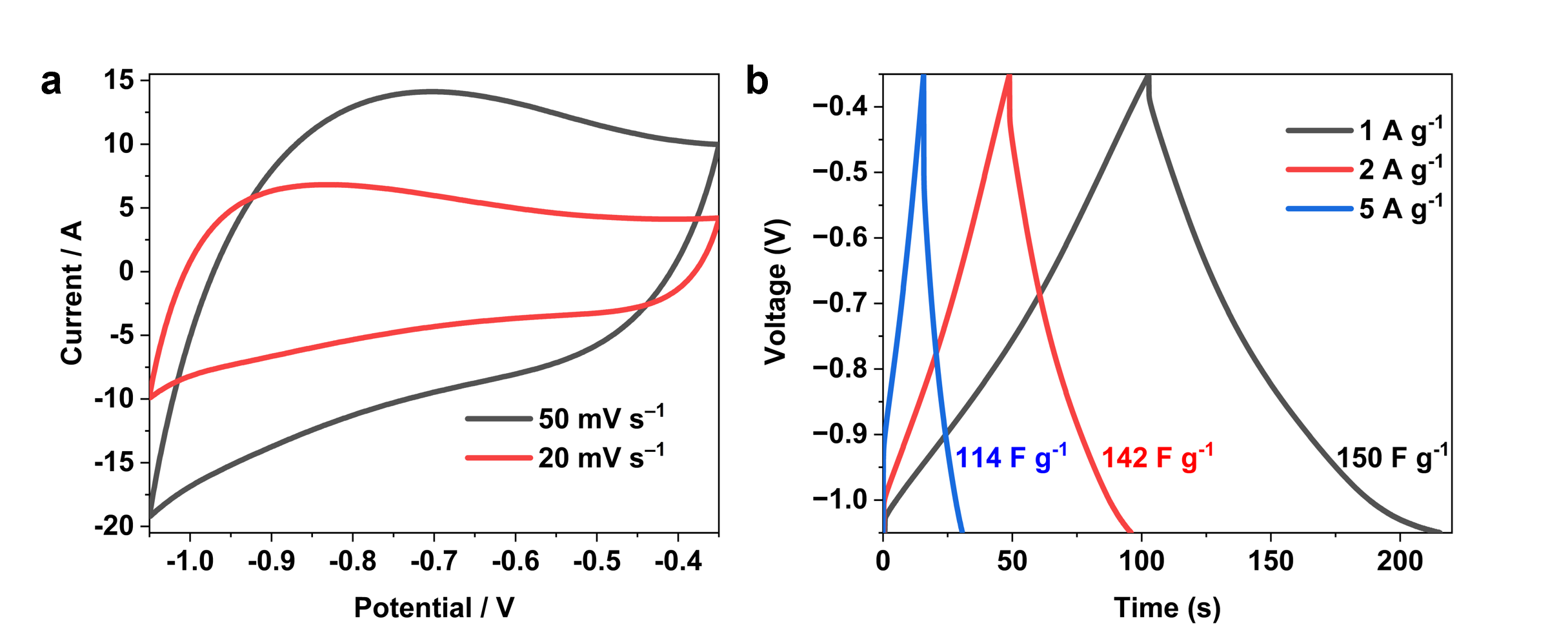

Supplement: Supplementary 1 — Figs. S1 to S5 Movie S1 [file research.0379.f1.zip › Fig. S4.tif]

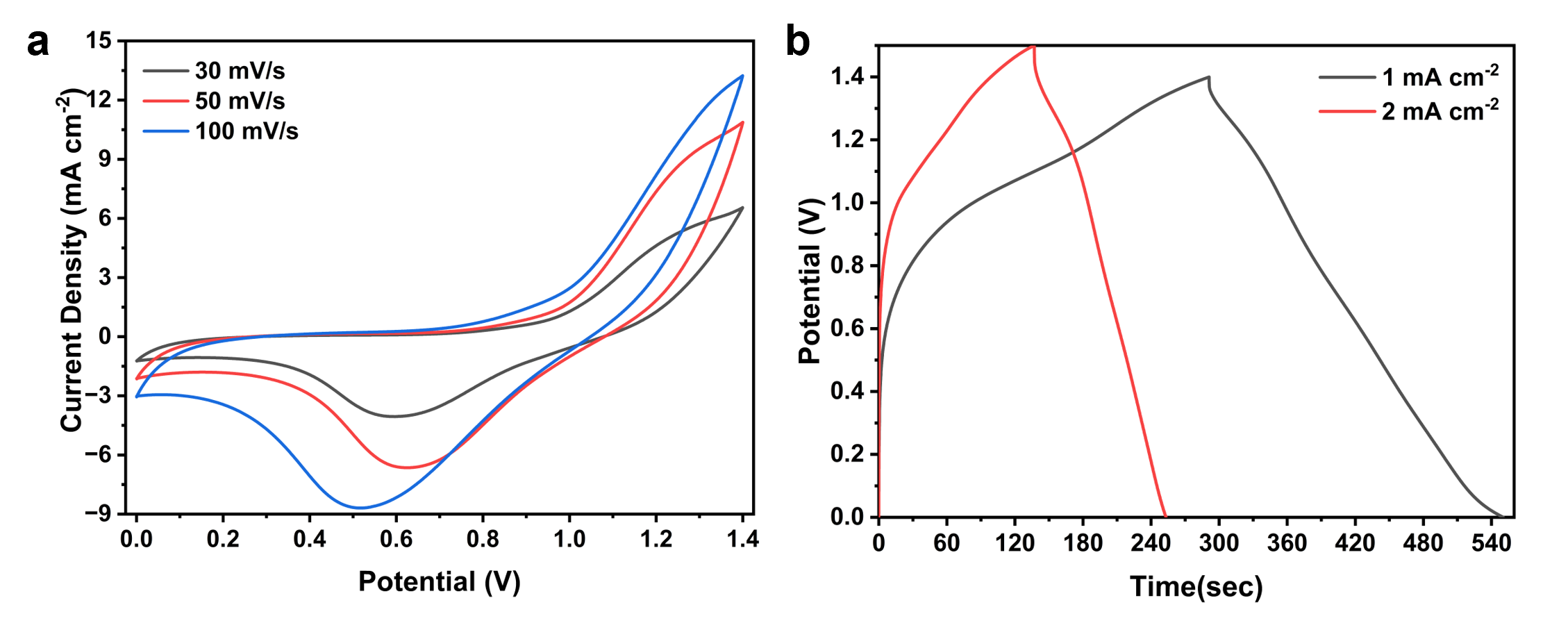

Supplement: Supplementary 1 — Figs. S1 to S5 Movie S1 [file research.0379.f1.zip › Fig. S5.tif]
